# Association of Prenatal Acetaminophen Exposure Measured in Meconium With Adverse Birth Outcomes in a Canadian Birth Cohort

**DOI:** 10.3389/fped.2022.828089

**Published:** 2022-04-05

**Authors:** Brennan H. Baker, Heather H. Burris, Tessa R. Bloomquist, Amélie Boivin, Virginie Gillet, Annie Larouche, Larissa Takser, Jean-Philippe Bellenger, Jean-Charles Pasquier, Andrea A. Baccarelli

**Affiliations:** ^1^Department of Environmental Health Sciences, Mailman School of Public Health, Columbia University, New York, NY, United States; ^2^Department of Pediatrics, Perelman School of Medicine, Children’s Hospital of Philadelphia, University of Pennsylvania, Philadelphia, PA, United States; ^3^Département de Pédiatrie, Faculté de Médecine et des Sciences de la Santé, Université de Sherbrooke, Sherbrooke, QC, Canada; ^4^Département de Psychiatrie, Faculté de Médecine et des Sciences de la Santé, Université de Sherbrooke, Sherbrooke, QC, Canada; ^5^Département de Chimie, Faculté des Sciences, Université de Sherbrooke, Sherbrooke, QC, Canada; ^6^Département d’Obstétrique et Gynécologie, Faculté de Médecine et des Sciences de la Santé, Université de Sherbrooke, Sherbrooke, QC, Canada

**Keywords:** paracetamol, birthweight, gestational age, maternal effects, meconium, development origins of health and disease (DOHaD)

## Abstract

**Background:**

The small number of studies examining the association of prenatal acetaminophen with birth outcomes have all relied on maternal self-report. It remains unknown whether prenatal acetaminophen exposure measured in a biological specimen is associated with birth outcomes.

**Objectives:**

To investigate the association of acetaminophen measured in meconium with birthweight, gestational age, preterm birth, size for gestational age, gestational diabetes, preeclampsia, and high blood pressure.

**Methods:**

This birth cohort from Sherbrooke, QC, Canada, included 773 live births. Mothers with no thyroid disease enrolled at their first prenatal care visit or delivery. Acetaminophen was measured in meconium for 393 children at delivery. We tested associations of prenatal acetaminophen with birthweight, preterm birth, gestational age, small and large for gestational age, gestational diabetes, preeclampsia, and high blood pressure. We imputed missing data *via* multiple imputation and used inverse probability weighting to account for confounding and selection bias.

**Results:**

Acetaminophen was detected in 222 meconium samples (56.5%). Prenatal acetaminophen exposure was associated with decreased birthweight by 136 g (β = −136; 95% CI [−229, −43]), 20% increased weekly hazard of delivery (hazard ratio = 1.20; 95% CI [1.00, 1.43]), and over 60% decreased odds of being born large for gestational age (odds ratio = 0.38; 95% CI [0.20, 0.75]). Prenatal acetaminophen was not associated with small for gestational age, preterm birth, or any pregnancy complications.

**Conclusion:**

Prenatal acetaminophen was associated with adverse birth outcomes. Although unobserved confounding and confounding by indication are possible, these results warrant further investigation into adverse perinatal effects of prenatal acetaminophen exposure.

## Introduction

Acetaminophen (also known as paracetamol) is the only analgesic recommended by doctors for pregnant women, as prenatal exposure to other drugs commonly used to treat pain and fever such as aspirin and non-steroidal anti-inflammatory drugs (e.g., ibuprofen and indomethacin) have previously been associated with birth defects, premature ductus arteriosus closure, and miscarriage (1–8). Accordingly, acetaminophen is the most commonly used over-the-counter pain medication taken during pregnancy, with use reported by over half of pregnant women in many populations (9, 10). During the last two decades, however, research from a multitude of diverse birth cohort studies has revealed consistent associations of prenatal acetaminophen exposure with adverse childhood outcomes including asthma, attention deficit hyperactivity disorder (ADHD), and autism (11–13). The severity of these adverse outcomes combined with such high rates of prenatal acetaminophen exposure makes further research an urgent public health priority. Indeed, a recent consensus statement supported by 91 scientists, clinicians and public health professionals from across the globe calls on health professionals to caution against the indiscriminate use of acetaminophen during pregnancy (14).

One possibility is that the development of childhood disorders associated with prenatal acetaminophen exposure may be mediated *via* adverse birth outcomes, such as reduced birthweight and preterm birth. For instance, birth cohort studies have shown associations of pre-pregnancy and prenatal acetaminophen exposure with low birthweight (15) and preterm birth (10), respectively, and large meta-analyses have shown associations of low birthweight and preterm birth with asthma (16, 17), ADHD (18, 19), and autism (20, 21). However, these adverse outcomes may also be explained by risk factors and indications for acetaminophen use during pregnancy, such as tobacco use, obesity, headaches, pain, fever, and infections (22).

The limited number of studies reporting associations of prenatal acetaminophen with birth outcomes may be a consequence of inaccurate exposure assessment. To the best of our knowledge, all but two cohort studies investigating the effects of prenatal acetaminophen exposure on children’s health have relied on mothers to self-report their acetaminophen use during pregnancy (23, 24). Furthermore, the only studies to show associations of prenatal acetaminophen with birth outcomes administered questionnaires during pregnancy and postpartum to assess maternal acetaminophen use (10, 15). Consequently, adverse birth outcomes could have influenced maternal responses in the postpartum interviews; when outcomes are suboptimal, women might be more likely to recall any potential explanatory behavior (25–27). Self-reported exposure assessment could also result in misclassification bias toward the null.

The possibility of misclassification bias can be eliminated by measuring prenatal acetaminophen exposure in a biological sample rather than relying on maternal self-report. Measuring chemicals in meconium, the first feces of newborn infants, has proven to be an effective, non-invasive method to assess cumulative prenatal exposures (24, 28, 29). Chemicals in meconium are known to have passed through the fetus and into the fetal intestinal tract (30–33), making meconium an ideal substrate for measuring *in utero* exposures. Furthermore, meconium measurements reflect cumulative exposures during the 2nd and 3rd trimesters of pregnancy, as xenobiotics and their metabolites are deposited in meconium throughout that period (30–33). Approximately 12% of all deliveries show evidence of meconium stained amniotic fluid, indicating that meconium was passed *in utero* (34). Whether meconium is passed *in utero* is likely non-random: risk factors for meconium stained amniotic fluid include advanced gestational age and prolonged labor (35). It is therefore important to account for selection bias when relying on exposures measured in this substrate. The primary aim of this study was to evaluate the association of acetaminophen measured in meconium with birth outcomes and pregnancy complications using inverse probability weighting methods to account for confounding and selection bias.

## Materials and Methods

### Study Population

**FIGURE 1:**
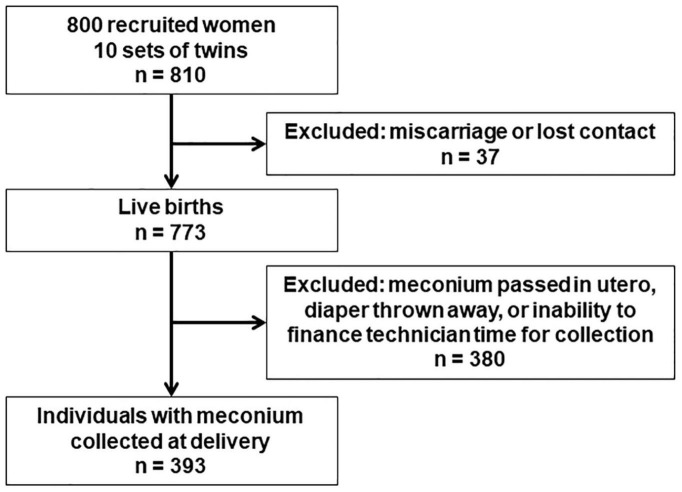
Gestation and the environment (GESTE) cohort flowchart.

This analysis was conducted in the GESTation and the Environment (GESTE) cohort in Sherbrooke, QC, Canada. The cohort was initially designed to examine the effects of environmental contaminants on endocrine disruption. Women age ≥ 18 years without chronic medical conditions and with no known thyroid disease enrolled at the Research Center of the CHUS (Centre Hospitalier Universitaire de Sherbrooke) from September 25, 2007, to September 10, 2009. Half of the cohort was enrolled at the beginning of pregnancy at their first prenatal care visit, and the other half was enrolled at delivery. Recruitment at delivery excluded very preterm births before 33 weeks completed gestation. Among the 800 women recruited, 37 were excluded due to loss to follow up or miscarriage and 10 gave birth to twins, resulting in 773 live births (Figure 1). All study protocols were approved by the institutional review boards of the University of Sherbrooke and Columbia University.

### Exposure

Meconium was collected from diapers of infants after delivery and stored within 10 mins at −80°C until analysis, which occurred in 2017–2018. Acetaminophen was extracted from <120 mg meconium and analyzed with ultraperformance liquid chromatography mass spectrometry following the methods described elsewhere (28). Among all 773 live births, the eligible study sample was 393 individuals for whom meconium was collected at delivery (Figure 1). Meconium was not always collected, for instance if the infant passed it *in utero*, if the diaper was thrown in the trash before the technician was able to collect it, or if we were unable to finance technician time for collecting the sample. These potential sources of selection bias were addressed by weighting on the inverse probability of selection (see statistical analysis for details). Acetaminophen was measured with a recovery of 104% and repeatability of ±15%. The limit of detection (LOD) and limit of quantification (LOQ) were 2 and 5 ng/g, respectively.

### Outcome

Data for birthweight and gestational age were obtained from CHUS medical records. Infants were weighed on Scale-tronix pediatrics scale 4802 by the obstetric team. Preterm birth was defined as birth before 37 completed weeks of gestation. Small for gestational age (SGA) and large for gestational age (LGA) were defined as birthweights below the 10th percentile and above the 90th percentile for gestational age, respectively. We assigned SGA and LGA categories and computed birthweight for gestational age z-scores in accordance with the Fenton international growth chart (36). Data on pregnancy complications, including gestational diabetes, preeclampsia, and high blood pressure, were obtained from CHUS medical records. Complete birth outcome data were available for all 393 individuals with meconium collected at delivery.

### Statistical Analysis

Covariate data were obtained from CHUS medical records and questionnaires administered after delivery. Covariates were child sex, familial income, and maternal characteristics including age at delivery, education status (College/University vs. no College/University), pre-pregnancy BMI, smoking during pregnancy (yes/no), and alcohol during pregnancy (yes/no).

Differences in baseline characteristics between (1) individuals with and without prenatal acetaminophen exposure, and (2) individuals with and without meconium collected at delivery were determined using chi-square goodness of fit tests for binary variables and two-sample t-tests for continuous variables. Using linear regression, we estimated associations of meconium acetaminophen detection (yes vs. no) with birthweight in grams and birthweight for gestational age z-score. Using logistic regression, we estimated associations of meconium acetaminophen detection with SGA, LGA, preterm birth, and maternal gestational diabetes, preeclampsia, and high blood pressure. Using cox proportional hazards models with birth as the event and gestational age as the time to event, we estimated the association of meconium acetaminophen detection with the hazard for giving birth. Because birth (the event) occurred for all individuals, increased hazards in these models indicate shorter gestational age (the time to event). Confidence intervals for cox models were calculated with robust standard errors. Coefficients from logistic regressions and cox models were exponentiated into odds ratios and hazard ratios, respectively. To account for missing covariate data, all models were employed on 10 datasets imputed using the “MICE” R package (37). Estimates and standard errors from imputed datasets were combined using Rubin’s Rule (38, 39).

In addition to unadjusted models, we report models adjusted for the covariates described above. We controlled for covariates by inverse probability of exposure weighting (IPW) using propensity scores (40–43). Propensity scores (*p*, the likelihood of detectable meconium acetaminophen) were estimated using logistic regression models in which exposure (meconium acetaminophen detected vs. not detected) was regressed on the covariates described above. Weights were estimated as 1/*p* for exposed individuals, and 1/(1 – *p*) for unexposed individuals. Covariate balancing propensity scores and weights were computed using the “CBPS” R package (44). This study sample weighting on the inverse probability of exposure creates a pseudo-population balanced on measured baseline covariates (40, 41, 43). In a sensitivity analysis to assess the impact of recruitment at the start of pregnancy versus delivery, we reconstructed these covariate adjusted models stratified by time of recruitment.

We additionally addressed potential selection bias related to meconium collection by weighting models on both the inverse probability of exposure described above and the inverse probability of selection. We first created weights for the probability of selection using logistic regression models in which selection (meconium collected vs. not collected) was regressed on the covariates described above for the entire 810 individual cohort (800 recruited women, 10 sets of twins). Then we removed those without meconium data and created weights for the probability of exposure as described above before fitting weighted models of the effect of meconium acetaminophen exposure on birth outcomes. Weighting the 393 individual selected sample on the probability of selection creates a pseudo-population that is comparable on measured covariates to the entire 810 individual cohort.

We computed E-values to assess the potential for unmeasured confounding (45). E-values indicate the minimum strength of association, on the risk ratio scale, of an unmeasured confounder with both the exposure and outcome that would confound a null effect to the observed effect estimate (i.e., completely explain the observed association between the exposure and outcome). In calculating E-values, all effects are converted to the risk ratio scale, with continuous outcomes dichotomized based on the exposure effect size on the outcome (Cohen’s d). E-values were calculated for birth outcomes that were significantly associated with meconium acetaminophen based on covariate adjusted models. Statistical analyses were conducted with R, version 3.5.1 (46).

## Results

Baseline covariates stratified by meconium acetaminophen detection are presented in Table 1. Acetaminophen was detected in the meconium of 222 individuals (56.5%) among the total study sample of 393 (Table 1). Among those with quantifiable acetaminophen levels, the geometric mean (geometric SD) concentration of acetaminophen was 123.8 [9.8] ng/g.

**TABLE 1:** Characteristics of study sample stratified by detection of acetaminophen in meconium in the Gestation and the environment (GESTE) cohort (*n* = 393).

	No acetaminophen (*N* = 171)	Acetaminophen (*N* = 222)	Total (*N* = 393)	*P* Value
Sex				0.968
Female	82 (48.0%)	106 (47.7%)	188 (47.8%)	
Male	89 (52.0%)	116 (52.3%)	205 (52.2%)	
Maternal age at delivery (years)				0.986
Mean (SD)	28.9 (4.7)	28.9 (4.5)	28.9 (4.6)	
Range	18.0 - 43.0	19.0 - 41.0	18.0 - 43.0	
Maternal education				0.980
No College or University	68 (39.8%)	88 (39.6%)	156 (39.7%)	
College or University	103 (60.2%)	134 (60.4%)	237 (60.3%)	
Family income (Canadian dollars)				0.596
N-Miss	21	18	39	
Mean (SD)	69,874 (47,968)	67,235 (44,858)	68,353 (46,153)	
Range	2,600 – 500,000	8000 – 450,000	2,600 – 500,000	
Maternal BMI (kg/m^2^)				0.020
N-Miss	0	1	1	
Mean (SD)	24.9 (5.2)	26.2 (5.9)	25.7 (5.6)	
Range	17.9 - 45.2	17.7 - 49.1	17.7 - 49.1	
Smoked during pregnancy				0.927
N-Miss	8	5	13	
No	141 (86.5%)	187 (86.2%)	328 (86.3%)	
Yes	22 (13.5%)	30 (13.8%)	52 (13.7%)	
Alcohol during pregnancy				0.161
N-Miss	8	5	13	
No	120 (73.6%)	173 (79.7%)	293 (77.1%)	
Yes	43 (26.4%)	44 (20.3%)	87 (22.9%)	

Infants with acetaminophen detected in meconium had a mean (range) birthweight of 3,338 g (1,825, 4,700) and mean gestational age of 39.0 weeks (33, 41) compared with 3,459 g (1,785, 4,705) and 39.3 weeks (35, 41) for unexposed infants (Table 2). Accounting for all covariates, prenatal acetaminophen exposure was associated with decreased birthweight by 136 grams (β = −136; 95% CI [−229, −43]) and decreased birthweight for gestational age z-score (β = −0.17; 95% CI [−0.34, 0.00]) (Table 2). Consistent with these associations with decreased birthweight, prenatal acetaminophen was also associated with over 60% decreased odds of LGA (odds ratio [OR] = 0.38; 95% CI [0.20, 0.75]) (Table 2). In addition to their decreased birthweight and lower likelihood of LGA, the mean time of gestation was on average 0.3 weeks shorter among individuals exposed to acetaminophen *in utero* compared to the unexposed group (39.0 vs. 39.3 weeks). Accordingly, prenatal acetaminophen exposure was associated with a 20% increased weekly hazard of delivery (hazard ratio = 1.20; 95% CI [1.00, 1.43]), indicating a higher likelihood for earlier delivery and thus reduced gestational age in exposed individuals (Table 2). Our data suggest that prenatal acetaminophen was not associated with SGA or preterm birth (Table 2). Results were similar in a covariate adjusted sensitivity analysis stratified by recruitment (Supplementary Table 1). For outcomes that were significantly associated with meconium acetaminophen in the main analysis, the direction of the association was the same in both strata, although the magnitude of the associations differed between women recruited at the start of pregnancy versus delivery in some cases. For instance, the association of meconium acetaminophen with reduced birthweight was stronger for women recruited during pregnancy compared to women recruited at delivery.

**TABLE 2:** Association of meconium acetaminophen with birth outcomes (*N* = 393).

	Mean (range) or *n* (%)		Coefficient, hazard ratio, or odds ratio^a^		
	Acetaminophen	No acetaminophen	Unadjusted	Covariate adjusted^b^	Selection bias adjusted ^c^
Birthweight (g)	3,338	3,459	−121	−136	−141
	(1,825, 4,700)	(1,785, 4,705)	[−213, −28]	[−229, −43]	[−232, −49]
Birthweight for gestational age z-score	−0.129	0	−0.13	−0.17	−0.18
	(−2.57, 2.14)	(−1.97, 2.42)	[−0.3, 0.04]	[−0.34, 0]	[−0.35, −0.01]
Gestational age (weeks)	39	39.3	1.23	1.2	1.21
	(33, 41)	(35, 41)	[1, 1.5]^d^	[1, 1.43]^d^	[1.01, 1.45]^d^
Small for gestational age	15 (6.8)	13 (7.6)	−0.13	−0.08	0.01
			[−0.9, 0.64]	[−0.63, 0.46]	[−0.37, 0.39]
Large for gestational age	7 (3.2)	12 (7.0)	0.43	0.38	0.36
			[0.17, 1.12]	[0.2, 0.75]	[0.22, 0.58]
Preterm birth	8 (3.6)	9 (5.3)	0.67	0.7	0.62
			[0.25, 1.78]	[0.35, 1.41]	[0.38, 1.02]

*^a^Coefficients from linear regression shown for birthweight and birthweight for gestational age z-score. Hazard ratios from cox proportional hazard models shown for gestational age. Odds ratios from logistic regression shown for small for gestational age (SGA), large for gestational age (LGA), and preterm birth.*

*^b^Adjusted for covariates by inverse probability of exposure weighting using child sex, familial income, and maternal age, education, BMI, smoking during pregnancy, and alcohol during pregnancy to predict exposure.*

*^c^Adjusted for covariates by inverse probability of exposure weighting and for selection bias by weighting on the inverse of the probability of selection.*

*^d^Ratio for instantaneous hazard of delivery.*

We conducted sensitivity analyses for selection bias and unmeasured confounding. Mothers of children for whom meconium was collected were significantly older and more educated compared to those without meconium data (Supplementary Table 2), indicating the potential for selection bias. Controlling for selection bias by weighting models to account for these covariate differences, however, did not appreciably impact the estimates of the effects of meconium acetaminophen detection on any birth outcome, suggesting minimal bias related to meconium sampling in this cohort (Table 2). Sensitivity analyses for unmeasured confounding show that an unmeasured confounder would need to increase the risk of both prenatal acetaminophen exposure and low birthweight by 93% in order to explain away the association of prenatal acetaminophen with reduced birthweight (E-value = 1.93, Supplementary Table 3). E-values were greater than 1.5 for all other birth outcomes that were significantly associated with meconium acetaminophen (Supplementary Table 3).

Meconium acetaminophen was not associated with pregnancy complications including gestational diabetes, preeclampsia, or high blood pressure (Table 3).

**TABLE 3:** Associations of meconium acetaminophen with pregnancy complications (*N* = 393).

	Mean or *n* (%)		Odds ratio		
	Acetaminophen	No acetaminophen	Unadjusted	Covariate adjusted^a^	Selection bias adjusted^b^
Gestational diabetes	30 (13.5)	20 (11.7)	1.18 [0.64, 2.16]	1.05 [0.69, 1.6]	1.02 [0.76, 1.37]
Preeclampsia	3 (1.4)	2 (1.2)	1.16 [0.19, 7.01]	1.01 [0.29, 3.53]	0.88 [0.36, 2.16]
High blood pressure	19 (8.6)	11 (6.4)	1.36 [0.63, 2.94]	1.08 [0.64, 1.83]	1.13 [0.77, 1.64]

*^a^Adjusted for covariates by inverse probability of exposure weighting using child sex, familial income, and maternal age, education, BMI, smoking during pregnancy, and alcohol during pregnancy to predict exposure.*

*^b^Adjusted for covariates by inverse probability of exposure weighting and for selection bias by weighting on the inverse of the probability of selection.*

## Discussion

### Principal Findings

In this Eastern Canadian birth cohort, detection of acetaminophen in meconium, which may indicate prenatal acetaminophen exposure during the 2nd and 3rd trimesters of pregnancy, was likely associated with decreased birthweight, decreased gestational age, and decreased odds of LGA. Although meconium was only collected for approximately half of our eligible births, we found no evidence that selection bias impacted effect estimation: associations of meconium acetaminophen with outcomes were nearly identical in both models adjusted and not adjusted for selection bias.

### Strengths

Our study has several strengths. First, the high genetic and sociodemographic homogeneity in the GESTE cohort limits the likelihood of confounding by unknown genetic or sociodemographic factors. Second, the prospective nature of the cohort limits sources of bias common in retrospective designs, including selection and recall bias. Third, we explicitly controlled for known sources of confounding and selection bias. Finally, our measurement of prenatal acetaminophen exposure in meconium eliminates the possibility of recall bias.

### Limitations

This study has limitations. First, while the homogeneity of the GESTE cohort may limit confounding, it also lowers the generalizability of results. Even within our own study, the magnitude of the association of meconium acetaminophen with birthweight was not entirely generalizable between individuals recruited during pregnancy and at delivery. This could be explained by differing distributions of unmeasured genetic and environmental effect modifiers in each group. Second, this study had a relatively small sample size of just under 400 mother child pairs. Consequently, there were few events for several outcomes, including early prematurity and severe preeclampsia. Third, our conclusions regarding preterm birth may be limited by the exclusion of some extremely preterm deliveries. Although unlikely, this could be a source of selection bias if there are different effects of prenatal acetaminophen exposure on the risk for preterm birth before 33 weeks versus before 37 weeks. Fourth, we lacked the information necessary to control for indications for acetaminophen use, such as chronic pain, fever, and infections during pregnancy. However, when controlling for indications for acetaminophen in the Danish National Birth Cohort study, Rebordosa and colleagues still observed associations of prenatal acetaminophen with increased risk of preterm birth (10). However, results from other cohorts may not be generalizable to this Eastern Canadian population. Therefore, confounding by indication remains a possibility. Fifth, it is possible that acetaminophen was more easily detected in the meconium of smaller infants owing to less efficient metabolism. However, acetaminophen pharmacokinetics in the fetus parallels that in the mother, with fetal and maternal acetaminophen reaching comparable levels as early as 30 mins after maternal administration (47). Inverse causality is thus unlikely. Sixth, we did not ask women to self-report their use of acetaminophen during pregnancy, so we were unable to correlate acetaminophen intake with levels of acetaminophen in meconium. Thus, while detection of acetaminophen in meconium may indicate some level of exposure during the 2nd and 3rd trimesters of pregnancy, this measure does not disclose either the duration or dose of exposure. Future studies are needed to determine the dosage and timing of acetaminophen required to have detectable levels in meconium, and at what range of exposures adverse outcomes may occur. Seventh, since meconium does not form until the later stages of pregnancy, its utility as a substrate for measuring prenatal exposures may be limited for studies of severe prematurity and early pregnancy outcomes. Finally, meconium samples were stored at −80°C for approximately 10 years before analysis. Several studies have shown high stability of acetaminophen in human biospecimens, including plasma and urine, after storage at −20 or −80°C for 3–6 months, and through multiple freeze-thaw cycles (48–51). However, our samples were stored for longer, and the effect of storage conditions on the stability of acetaminophen in meconium has not been studied. Thus, sample degradation remains a possibility.

### Interpretation

A small number of studies have previously shown associations between maternal self-reported acetaminophen use during pregnancy and adverse birth outcomes. In the Danish National Birth Cohort, there was an increased risk of preterm birth among women using acetaminophen during the third trimester of pregnancy, but there were no associations of acetaminophen use with miscarriage, stillbirth, low birthweight, or SGA, or with common preterm birth complications including bronchopulmonary dysplasia, intracranial hemorrhage, retinopathy of prematurity, perinatal infections and anemia of prematurity (10). In the Ontario Birth Study, maternal acetaminophen use in the 3 months before pregnancy was associated with low birthweight and increased risk for SGA, but maternal acetaminophen use during pregnancy was not (15). However, both of those studies were prone to substantial recall bias, as they assessed maternal acetaminophen use *via* questionnaires administered during pregnancy and postpartum. When self-report occurs in postpartum interviews, mothers of infants with adverse birth outcomes may rack their brains for an explanation, thereby overreporting exposures (25–27). Our study, on the other hand, utilized a direct measurement of prenatal acetaminophen exposure measured in meconium that is unbiased by inaccurate recall.

While the associations of prenatal acetaminophen exposure with adverse birth outcomes found here may be concerning, more studies in a diverse range of cohorts are needed before suggesting a change in clinical practice. Additionally, evolving patterns of both antenatal management and acetaminophen use in this population since the study began in 2007 likely impacts the relevance of our results. For instance, one study found a decline in acetaminophen use during pregnancy by 2.5% for each 2-year period between 2004 and 2018 (22).

Mechanisms underlying the associations of prenatal acetaminophen exposure with adverse birth outcomes remain unknown. Acetaminophen may inhibit prostacyclin synthesis and thereby promote pre-eclampsia (52), which has previously been associated with intrauterine growth restriction and reduced gestational age (53). Acetaminophen exposure may also trigger the immune system and upregulate oxidative stress response pathways (54) that may underlie adverse birth outcomes. A better understating of the mechanisms through which prenatal acetaminophen exposure may affect birth outcomes is needed, not only to better assess causality, but also to serve as potential targets in future intervention studies.

## Conclusion

While this study may add evidence in support of questioning the safety of acetaminophen use during pregnancy, more work is needed to rule out confounding by indication and to assess generalizability before a change in clinical practice is recommended.

## Data Availability Statement

The original contributions presented in the study are publicly available. This code can be found here: https://github.com/brennanhilton/acetaminophen-birth-outcomes.

## Ethics Statement

The studies involving human participants were reviewed and approved by Institutional review boards of the University of Sherbrooke and Columbia University. Written informed consent to participate in this study was provided by the participants’ legal guardian/next of kin.

## Author Contributions

BB conceptualized and carried out the analyses, drafted the initial manuscript, reviewed, and revised the manuscript. HB conceptualized the analyses, reviewed, and revised the manuscript. TB, ABo, AL, and VG coordinated and supervised data collection, reviewed, and revised the manuscript. LT, J-PB, J-CP, and ABa designed the study, reviewed, and revised the manuscript. All authors contributed to the article and approved the submitted version.

## Conflict of Interest

The authors declare that the research was conducted in the absence of any commercial or financial relationships that could be construed as a potential conflict of interest.

## Publisher’s Note

All claims expressed in this article are solely those of the authors and do not necessarily represent those of their affiliated organizations, or those of the publisher, the editors and the reviewers. Any product that may be evaluated in this article, or claim that may be made by its manufacturer, is not guaranteed or endorsed by the publisher.
