# Supplementary material for: Association of Prenatal Acetaminophen Exposure Measured in Meconium With Adverse Birth Outcomes in a Canadian Birth Cohort
Source: Front Pediatr. 2022 Apr 5;10:828089. doi: 10.3389/fped.2022.828089 (PMC9017809; doi:10.3389/fped.2022.828089)
Supplement: Supplementary file 1 [file Table_1.DOCX]

Supplemental tables:

Supplemental Table 1: Covariate adjusted analysis stratified by time of recruitment (delivery versus pregnancy).

|  | Coefficient, Hazard Ratio, or Odds Ratio ^a^ | |
| --- | --- | --- |
| Outcome | Delivery (n = 257) | Pregnancy (n = 136) |
| Birthweight (g) | -123 [-234, -12] | -174 [-343, -5] |
| Birthweight for gestational age z-score | -0.15 [-0.36, 0.06] | -0.23 [-0.53, 0.07] |
| Gestational age (weeks) | 1.24 [0.99, 1.56] | 1.17 [0.86, 1.61] |
| Small for gestational age | 0.71 [0.38, 1.34] | 1.48 [0.49, 4.48] |
| Large for gestational age | 0.65 [0.24, 1.72] | 0.26 [0.1, 0.69] |
| Preterm birth | 0.64 [0.29, 1.43] | 0.64 [0.17, 2.41] |
| Gestational diabetes | 1.05 [0.64, 1.73] | 0.89 [0.42, 1.85] |
| Preeclampsia | 0.55 [0.09, 3.39] | 1.38 [0.24, 8.03] |
| High blood pressure | 1.94 [0.98, 3.85] | 0.4 [0.15, 1.12] |

^a^ Coefficients from linear regression shown for birthweight and birthweight for gestational age z-score. Hazard ratios from cox proportional hazard models shown for gestational age. Odds ratios from logistic regression shown for small for gestational age (SGA), large for gestational age (LGA), preterm birth, gestational diabetes, preeclampsia, and high blood pressure.

Supplemental Table 2: Characteristics of study sample stratified by meconium collection in the GESTation and the Environment (GESTE) cohort (n = 810).

|  | Meconium collected (N=393) | No meconium collected (N=417) | Total (N=810) | p value |
| --- | --- | --- | --- | --- |
| Sex |  |  |  | 0.429 |
| N-Miss | 0 | 37 | 37 |  |
| Female | 188 (47.8%) | 171 (45.0%) | 359 (46.4%) |  |
| Male | 205 (52.2%) | 209 (55.0%) | 414 (53.6%) |  |
| Maternal age at delivery |  |  |  | 0.004 |
| N-Miss | 0 | 33 | 33 |  |
| Mean (SD) | 28.91 (4.56) | 27.97 (4.62) | 28.45 (4.61) |  |
| Range | 18.00 - 43.00 | 18.00 - 41.00 | 18.00 - 43.00 |  |
| Maternal education |  |  |  | < 0.001 |
| No College or University | 156 (39.7%) | 227 (54.4%) | 383 (47.3%) |  |
| College or University | 237 (60.3%) | 190 (45.6%) | 427 (52.7%) |  |
| Family income (Canadian dollars) |  |  |  | 0.052 |
| N-Miss | 39 | 93 | 132 |  |
| Mean (SD) | 68353.67 (46153.47) | 62420.68 (30869.48) | 65518.44 (39675.38) |  |
| Range | 2600.00 - 500000.00 | 7000.00 - 180000.00 | 2600.00 - 500000.00 |  |
| Maternal BMI |  |  |  | 0.145 |
| N-Miss | 1 | 7 | 8 |  |
| Mean (SD) | 25.67 (5.64) | 25.06 (6.16) | 25.35 (5.91) |  |
| Range | 17.71 - 49.08 | 14.99 - 55.60 | 14.99 - 55.60 |  |
| Smoked during pregnancy |  |  |  | 0.163 |
| N-Miss | 13 | 73 | 86 |  |
| No | 328 (86.3%) | 284 (82.6%) | 612 (84.5%) |  |
| Yes | 52 (13.7%) | 60 (17.4%) | 112 (15.5%) |  |
| Alcohol during pregnancy |  |  |  | 0.406 |
| N-Miss | 13 | 73 | 86 |  |
| No | 293 (77.1%) | 274 (79.7%) | 567 (78.3%) |  |
| Yes | 87 (22.9%) | 70 (20.3%) | 157 (21.7%) |  |

Supplemental Table 3: E-value sensitivity analysis for unmeasured confounding

| Outcome | Cohen's d cutoff for dichotomized continuous outcome ^a^ | Risk ratio conversion ^b^ | E-value ^c^ |
| --- | --- | --- | --- |
| Birthweight (continuous) | -0.29 | 0.77 (0.64, 0.92) | 1.93 |
| Birthweight z-score (continuous) | -0.21 | 0.83 (0.69, 0.99) | 1.7 |
| Gestational age (hazard ratio for delivery) | NA | 1.12 (1.00, 1.28) | 1.52 |
| Large for gestational age (odds ratio) | NA | 0.38 (0.20, 0.75) | 4.64 |

^a^ Continuous outcomes are dichotomized based on the effect size (Cohen’s d), computed as the linear regression estimate divided by the standard deviation of the outcome.

^b^ All estimates are converted to risk ratios for calculation of the E-value. See VanderWeele (2017) for details and conversion formulae.

^c^ E-values indicate the minimum strength of association, on the risk ratio scale, of an unmeasured confounder with both the exposure and outcome that would completely explain the observed association between the exposure and outcome.
